# The extracellular matrix complexity of idiopathic epiretinal membranes and the bilaminar arrangement of the associated internal limiting membrane in the posterior retina

**DOI:** 10.1007/s00417-021-05156-6

**Published:** 2021-03-24

**Authors:** Annalisa Altera, Gian Marco Tosi, Marì Regoli, Elena De Benedetto, Eugenio Bertelli

**Affiliations:** 1 Department of Life Science, University of Siena, Siena, Italy; 2 Department of Molecular and Developmental Medicine, University of Siena, Siena, Italy; 3 Department of Medicine, Surgery and Neuroscience, University of Siena, Siena, Italy

**Keywords:** Vitreoretinal interface, Epiretinal membrane, Extracellular matrix, Laminin, Collagen IV, Internal limiting membrane

## Abstract

**Purpose:**

To study the composition of the internal limiting membrane (ILM) of the retina, the extracellular matrix (ECM) of idiopathic epiretinal membranes (iERMs), and the relationships occurring between the two membranes.

**Methods:**

Forty-six iERMs, 24 of them associated with the ILM, were collected and included in this study. The investigation has been carried out by immunofluorescence and confocal microscopy on glutaraldehyde- and osmium-fixed epon-embedded samples and on frozen samples. Sections were double or triple labelled with antibodies against vimentin; collagens I, III, IV, α5(IV), and VI; laminin 1 + 2; laminin α2-, α4-, α5-, β1-, β2-, β3-, γ1-, and γ2-chains; entactin; and fibronectin.

**Results:**

iERM thickness was not uniform. Almost 14% of iERMs showed thickenings due to folding of their ECM component under the cell layer. The vitreal side of iERMs was often shorter than the attached ILM. In this case, the ILM resulted folded under the iERM. ILMs contained laminin 111; laminin α2-, α5-, β1-, β2-, and γ1-chains; entactin; collagens I; α5(IV); [α1(IV)]_2_α2(IV); and VI. Laminins, entactin, and α5(IV) were gathered on the retinal half of the ILM, whereas collagens [α1(IV)]_2_α2(IV) and I were restricted to the vitreal side. Collagen VI was detected on both sides of the ILM. iERMs expressed laminin 111, collagens III, [α1(IV)]_2_α2(IV) and VI, entactin, and fibronectin. Entactin co-localized with laminins and collagen IV.

**Conclusions:**

Analysis of laminins and collagen chain expression indicates that ILM contains laminin 111 (former laminin 1), laminin 521 (former laminin 11), laminin 211 (former laminin 2), collagen [α1(IV)]_2_α2(IV), and collagen α3(IV)α4(IV)α5. In contrast, iERMs express only collagen [α1(IV)]_2_α2(IV) and laminin 111. In addition, both iERMs and ILMs contain entactin. The presence of three major constituents of the basement membranes co-localized together in iERMs is suggestive for a deranged process of basement membrane formation which fails to assemble properly. In view of the many interactions occurring among its proteins, the ECM of either the iERMs or the ILMs can account for their reciprocal adhesiveness. In addition, the peculiar deposition of the ECM observed in some samples of iERM is suggestive for its involvement in the formation of macular puckers.

**Supplementary Information:**

The online version contains supplementary material available at 10.1007/s00417-021-05156-6.

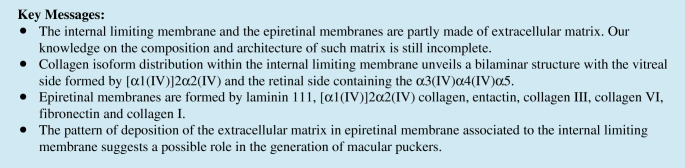



## Introduction

The internal limiting membrane (ILM) is the innermost layer of the retina. It is a thin acellular sheet of extracellular matrix (ECM) proteins which mediates the relationships between retina and vitreous [1]. The main constituents identified so far are collagens IV, VI, and XVIII; laminin; fibronectin; many proteoglycans; and hyaluronan [2–5], but proteomic analysis showed a broad variety of less expressed molecules [6]. The ILM is formed by the basal lamina of Müller cells and by some components of the vitreous blended with it [7]. Though from a structural point of view, it does not show any apparent complexity; the vitreoretinal interface is of critical importance as the site where a number of ocular pathologies develop including posterior vitreous detachment, macular hole, vitreomacular traction, idiopathic, and secondary epiretinal membranes (ERMs) [8]. ERMs are fibrocellular sheets of tissue that, when located in front of the macula and retract, generate a condition known as macular pucker which can strongly affect vision [9]. The cellular component of ERMs has been investigated in many occasions [10–17]. Though several studies have analysed a battery of molecular markers, the ultimate source of cells is still a matter of debate since results often appear inconclusive and conflicting. Whichever the cell type of origin is, cells located in ERMs take on a myofibroblastic phenotype testified by α-smooth muscle actin (α-SMA) expression [10–17] and by their ability to deposit new ECM [16, 17].

The ECM of ERMs, on the other hand, has received comparatively less attention. Most of the work carried out so far has been focused on the gross identification of the molecular components which include collagens I to VI [16, 18–23], heparansulfate proteoglycans [18], laminin [18, 22, 23], fibronectin [21, 22, 24], tenascin [25, 26], and decorin [26]. Very little is known on the mutual relationships occurring among EMC proteins within ERMs, and investigations on this matter have started only recently showing that in idiopathic ERMs (iERMs), collagens I and IV have pretty constant relationships. With only few exceptions cells can be consistently found gathered on the side of the iERM that faces the vitreous camera [23].

In order to facilitate ERM removal and the associated vitrectomy, the employment of a number of enzymes have been proposed to partially hydrolyse the ECM [27, 28]. However, a successful outcome strictly depends on the presence of the proper ECM substrates. Hence, a detailed knowledge of the ECM proteins exposed to the proteolytic process in both the ERMs and the ILM is essential for the selection of the appropriate enzyme or to device engineered new classes of pharmacologic vitreolytic agents [29]. In this respect, much work is still needed for a detailed characterization of iERMs. For instance, the precise identification of the many non-fibrillary components of the ECM still appears largely incomplete. It has been shown that iERMs contain laminin and collagen IV [18, 22, 23]. However, laminin and collagen IV may occur in 16 and 3 isoforms respectively [30, 31] and it is still unknown which of them is expressed in iERMs.

The present study is aimed to achieve a deeper understanding of the ECM composition of iERMs and of the associated ILM in the posterior retina using a battery of antibodies in immunofluorescence and confocal microscopy, including chain-specific anti-laminin and anti-collagen IV antibodies.

## Materials and methods

### Patient samples

Patients affected by iERMs were subjected to primary 25-gauge pars plana vitrectomy by the same surgeon (G.M.T.) without intraocular complications at the Ophthalmology Section of Siena University Hospital, Siena, Italy. The study followed the principles of the Declaration of Helsinki, and an institutional review board approved the research. Patients were treated after having signed a consent form that explained the nature of the offered treatment, its potential risks, benefits, adverse effects, and possible outcomes. ERMs were stained according to surgeon preference and were excised using a Grieshaber Revolution forceps (Alcon Laboratories Inc., Fort Worth, TX). At the end of the procedure, fluid/air exchange was performed in all patient.

A total number of 46 iERMs, 24 of them associated with the ILM, collected between January 2014 and August 2020 were included in this study. In addition, 2 ILMs excised from eyes affected by macular hole were included as control samples of ILM not associated with the ERMs. Thirty-six iERMs, 15 of them associated with the ILM, and one ILM were processed as follows: membranes were fixed with 1.25% glutaraldehyde in 0.1M sodium cacodylate for 24 h at 4°C and postfixed in 1% OsO_4_ for 2 h at 4°C. After fixation, samples were dehydrated and embedded in Epon following standard procedures. Semithin sections (1-μm thick), cut from each block of resin with an ultramicrotome Ultrotome Nova (LKB, Bromma, Sweden), were placed on Superfrost slides. Ten iERMs (9 of them associated with the ILM) and one ILM were embedded in OCT and frozen in cold isopentane dipped into liquid nitrogen. Sections were cut, air dried, fixed in cold acetone at −20°C for 10 min as previously reported [32], and stored at −80°C until used for immunofluorescence experiments.

### Antibodies

The following primary antibodies were used: mouse anti-vimentin (clone V9) monoclonal antibody (code V6389) was from Sigma-Aldrich (Saint Louis, MO); monoclonal mouse anti-laminin α5 (clone CL3118) (code NBP2-42391) and rabbit anti-collagen I (code NB600-408) antibodies were from Novus Biologicals Europe (Abingdon, UK); goat anti-collagen IV (code 1340-01) was purchased from Southern Biotech (Birmingham, AL); rabbit anti-collagen VI (code ab6588), rabbit anti-collagen III (ab7778), and rabbit anti-laminin 1+2 (code ab7463) antibodies were obtained from Abcam (Cambridge, UK); mouse anti-laminin-2, α2 chain specific, monoclonal antibody (clone 5H2) was from Gibco BRL (Gaithersburg, MD); mouse anti-laminin-5, γ2 chain-specific, monoclonal antibody (clone 4G1, code M7262) was obtained from Dako (Glostrup, Denmark); mouse anti-laminin γ1 (clone 2E8, code MAB1920), mouse anti-laminin α4 (clone 6C3, code sc-130541), and mouse anti-laminin-5, γ2 chain-specific (clone D4B5, code MAB19562), monoclonal antibodies were obtained from Chemicon (Temecula, CA); rat anti-α5(IV) (NC1 domain) collagen monoclonal antibody (clone 5H2, code 7077) was purchased from Chondrex Inc. (Woodinville, WA); rat anti-laminin β1 (clone LT3, code sc-33709), mouse anti-laminin β2 (clone C4, code sc-59980), and mouse anti-laminin β3 (clone A-6, code sc-133178) monoclonal antibodies were from Santa Cruz (Dallas, TX); and goat anti-entactin antibody (code AF2570) and rabbit anti-fibronectin antibody (code GTX112794) were obtained from R&D Systems (Minneapolis, MN) and GeneTex Inc. (Irvine, CA) respectively.

The secondary antibodies, all double-labelling grade, were donkey TRITC-conjugated anti-goat IgG (code AP180R) from Chemicon (Temecula, CA), donkey Cy2-conjugated F(ab’)_2_ fragment anti-rabbit IgG (code 711-226-152), donkey Cy5-conjugated F(ab’)_2_ fragment anti-rabbit IgG (code 711-176-152), donkey Dy-Light 649-conjugated F(ab’)_2_ fragment anti-rat IgG (code 712-496-153), and donkey Cy5-conjugated F(ab’)_2_ fragment anti-mouse IgG (code 715-176-150) from Jackson Immunoresearch laboratories (Baltimore, PA).

### Confocal microscopy

Confocal microscopy was carried out on epon-embedded semithin sections and on frozen sections. Immunoreactions on epon-embedded sections were performed upon resin removal and antigen retrieval as previously reported [23]. In brief: epoxy resin was removed placing slides for 7 min in a 1:1 solution of ethanol and a saturated solution of sodium hydroxide in ethanol. After several washings in ethanol and water, sections were blocked for 30 min with 20% donkey serum and 0.3% Triton X-100. Heat-induced antigen retrieval was achieved in autoclave at 120°C in 0.1M Trizma-base (pH 9.0) for 10 min. After washing with PBS, free aldehydes were quenched with freshly prepared 0.2% sodium borohydride for 10 min. Then, sections, thoroughly washed with water and PBS, were incubated with two or three primary antibodies raised in different animals. Acetone-fixed frozen sections were directly processed for immunolabelling with the exception of reactions involving the anti-laminin α5 antibody that required a de-glycosylation protocol. De-glycosylation was achieved incubating sections with denaturating buffer (0.5% SDS and 40 mM DTT) for 3 h at room temperature and subsequently with 0.025 Units/ml of PNGase F (Sigma Aldrich, Saint Louis, MO) in PBS for 18 h at 37°C.

Positive immunoreactions with primary antibodies were unveiled with the appropriate secondary antibodies conjugated with Cy2, TRITC, and Cy5 or Dy-Light 649. Controls were carried out following the same procedures except for the omission of the primary antibody. Images were acquired with an LSM510 Zeiss confocal microscope with selective multitracking excitation.

## Results

### General iERM architecture

**Fig. 1:**
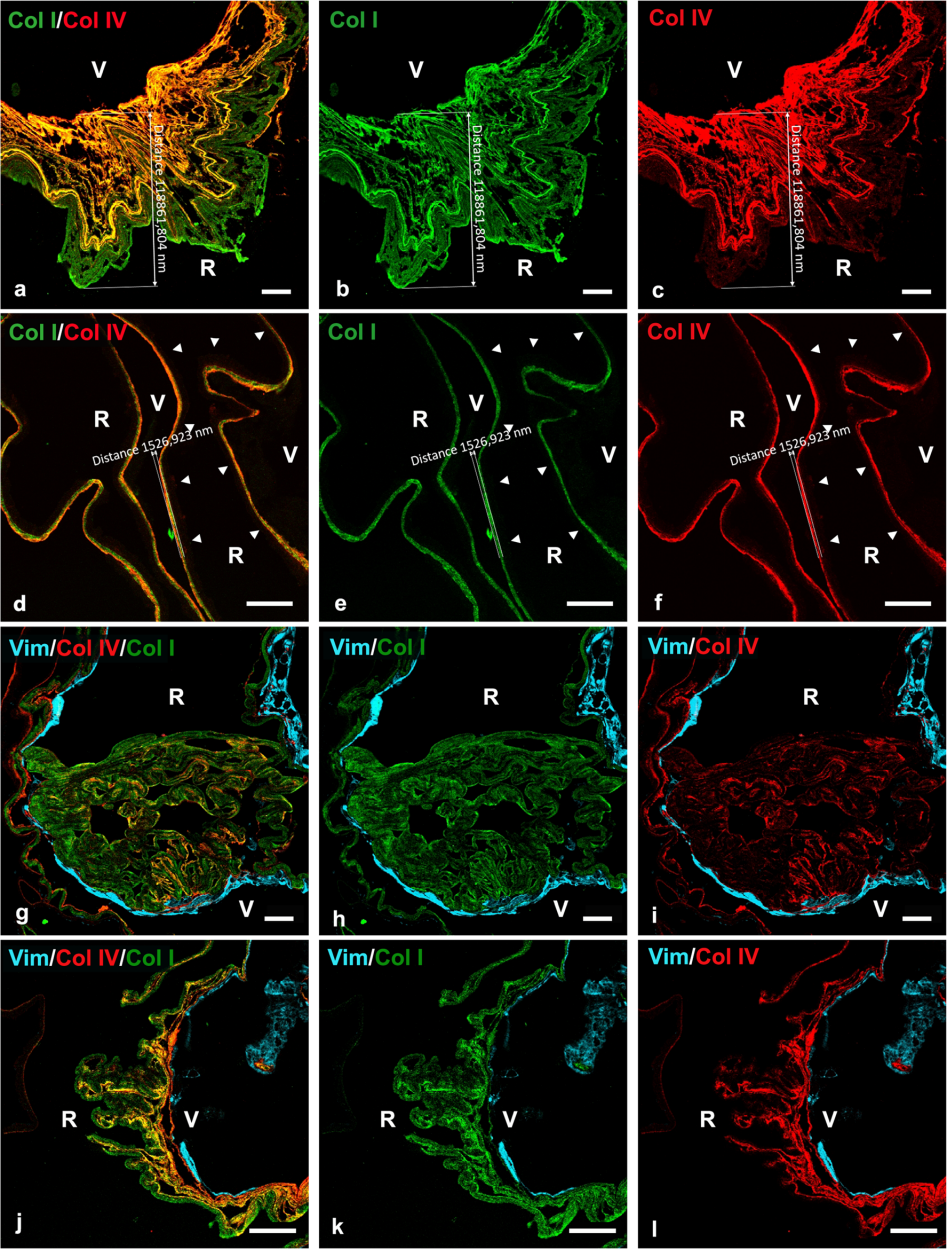
General arrangement of the ECM in iERMs. **a–f** Two iERMs, one of them associated with the ILM, double-labelled with anti-collagen I (green) and anti-collagen IV (red). **a–c** Idiopathic ERM provided with a very thick layer of ECM proteins. **d–f** Idiopathic ERM provided with a very thin layer of ECM proteins. The iERM has a closely associated ILM which is visible only for a very faint reddish background shadow (arrowheads). **g–l** Two iERMs triple-labelled with anti-vimentin (cyan), anti-collagen I (green) and anti-collagen IV (red) antibodies. **g–i** Knot-like local thickenings of the iERM, resulting from its folding under the cell layer, can be observed with a certain frequency. **j–l** Less pronounced foldings under the cell layer are likely knots-like accumulations in the process of formation. V vitreal side, R retinal side; magnification bars = 20 μm

The thickness of iERMs, as judged by collagen and cell staining on de-plasticized (epon de-embedded) sections, was extremely variable. Variability occurred between membranes but also within the same membrane so that in some areas iERMs could be extremely thin and in others much thicker. The average maximal and minimal thickness was 31.5±24.1 μm (range from 6.1 to 123 μm) and 2.6±2.4 μm (range from 0.5 to 11.1 μm) respectively. When considering exclusively the ECM, the average maximal and minimal thickness was respectively 23.2±23.9 (range from 1.6 to 123 μm) and 1.75±1.4 μm (range from 0.5 to 8.3 μm) (Fig. 1a–f). Focal thickenings, almost resembling membranous knots, could be observed in 5 samples (13.9% of cases). These thickenings were due to local folding of the ECM component of the membrane, and they looked as if formed by the sliding of the ECM under the cell layer (Fig. 1g–i). Less complex folding of the membranes could be possibly interpreted as knots in formation (Fig. 1j–l).

### Relationships between iERMs and ILM

**Fig. 2:**
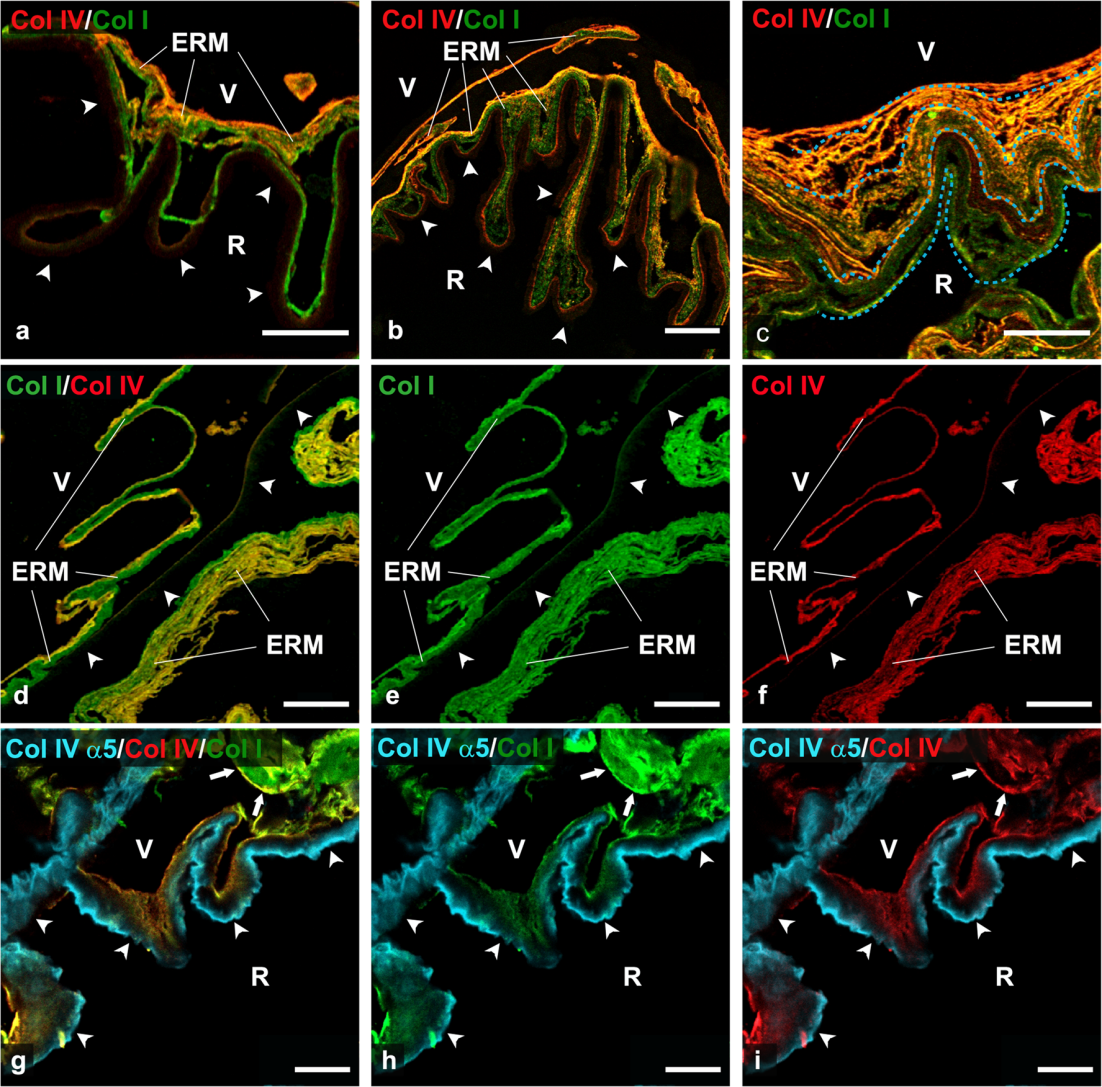
Relationships between iERMs and ILMs and differential distribution of collagen IV isoforms. **a–f** Four iERMs, three of them associated with the ILM, double-labelled with anti-collagen I (green) and anti-collagen IV (red). **g–i** One iERM associated with the ILM triple-labelled with the anti-α5(IV) (cyan), anti-collagen I (green) and anti-collagen IV (red). In addition to a perfectly parallel and adherent course, iERMs can display a more complex pattern of association with the ILM: **a** Idiopathic ERMs can be seen partially detached from the ILM (arrowheads), bridging different areas of the ILM. The result is the folding of the ILM under the iERM; **b** Idiopathic ERMs can adhere to the ILM (arrowheads) though their thickness is not uniform. The result is again the folding of the ILM. On the right side of **b**, the pattern of iERM/ILM association is similar to the sample shown in **a**, with a partially detached iERM. **c** In other cases, iERMs can have a lamellar and looser arrangement. In such circumstances, it is possible to observe the decreasing length of the collagen lamellae from the retinal side of the membrane towards the vitreal side. The progressive shortening of the lamellae is highlighted by the dotted line. **d–f** Idiopathic ERM partially associated with the ILM (arrowheads). The ILM is double-labelled on its vitreal rim (the one facing the iERM). **g–i** The iERM (arrows) and the vitreal rim of the ILM are double-labelled with anti-collagen IV and anti-collagen I antibodies. In contrast, the antibody against the α5-chain of collagen IV labels exclusively the retinal half of the ILM (arrowheads). The absence of co-localization between anti-α5(IV) and anti-collagen IV antibodies indicates that the latter antibody recognizes the ([α1(IV)]_2_α2(IV) isoform, the only isoform that does not contain the α5-chain. Where optimally cross-sectioned, ILM also shows an unlabelled stripe between the anti-collagen I/IV double labelled vitreal rim and the anti-α5(IV) immunoreactive retinal half ERM = Epiretinal membrane; V vitreal side; R retinal side. **a-f**) Magnification bars = 20 μm.** g-i**) Magnification bars = 10 μm.

When epon de-embedded sections contained both iERM and ILM, the relationships occurring between the two structures displayed three possible arrangements. The first type was observed when iERMs doubled the ILM following closely its course and matching exactly its length. In general, these relationships were characterized by very thin iERMs strongly adherent to the ILM (Fig. 1d–f). A second arrangement occurred when iERMs appeared attached to the ILM in a discontinuous way as previously reported [20]. However, iERMs were not simply detached from the ILM. Indeed, the length of the two membranes was not the same so that the ILM was longer and showed a winding course compared to the shorter and straighter course of the iERM. On the whole, in these cases, the iERM seemed to bridge distant points of the ILM (Fig. 2a). A closer look to this arrangement, however, showed that such relationships were probably due to a delamination process that must have occurred during iERM development. This was testified by the presence of a thin layer of collagen I which could be almost invariably found adherent to the whole length of the ILM and that likely resulted from iERM dissociation (Fig. 2a). The third type was a sort of intermediate arrangement in which, as for the second type, the length of the inner (vitreal) side of the iERM did not match with the longer ILM. However, in this instance, the iERM was continuously adherent to the ILM and the length mismatch was compensated by local thickenings of the ECM (Fig. 2b). As previously reported [23], collagens in iERMs frequently showed a loose lamellar arrangement where lamellae appeared partially detached one from the other. Because of such loose arrangement, it was easy to observe that collagen lamellae grew shorter proceeding from the outside (retinal side) inward (toward the vitreous) particularly in that portion of the membrane (vitreal side) where immunoreactivity for collagen IV was stronger (Fig. 2c).

### Immunofluorescence and confocal microscopy on de-plasticized sections

In order to further characterize the composition of iERMs, we tested a battery of antibodies against proteins of the ECM. As previously reported [23], collagens I and IV followed a constant pattern of distribution. The side of the iERM facing the ILM was characterized by a layer of collagen I of variable thickness, whereas the side facing the vitreous was formed by a layer of collagens I and IV mixed together. These findings are evident also in this study (Figs. 1 and 2). When portions of the ILM could be found isolated from the iERMs, its vitreal edge was stained with both anti-collagen IV and anti-collagen I antibodies though the bulk of the ILM remained unlabelled (Fig. 2d–f). To test whether collagen I and IV immunoreactivities actually belonged to the ILM or were due to a thin layer of the previously attached iERM left behind, we also stained an ILM that was removed from a hypermyopic eye with a macular hole. The sample that was not complicated by the presence of an iERM gave the same results confirming that the vitreal rim of the ILM contained both collagen IV and collagen I (Online Resource 1). Negative controls did not show any staining either for collagen I or collagen IV.

To detect specific laminin and collagen IV isoforms, we tested several antibodies on de-plasticized sections. Unfortunately, possibly because of the strong fixation protocols resistant to antigen retrieval procedures, we were not able to get any consistent result and we had to complete our observations on frozen samples.

### Immunofluorescence and confocal microscopy on frozen sections

Collagen IV is formed by 3 α-chains. Six different α-chains, α1(IV), α2(IV), α3(IV) α4(IV), α5(IV), and α6(IV), can be found in collagen IV. They assemble in three possible isoforms: [α1(IV)]_2_α2(IV), α3(IV)α4(IV)α5(IV), and [α5(IV)]_2_α6(IV). In order to determine which isoform of collagen IV is expressed in iERMs, we carried out triple labelling experiments with the same antibody successfully employed on de-plasticized sections along with the anti-collagen I and the anti-α5(IV) antibodies. The anti-α5(IV) antibody was chosen as the α5(IV) chain is shared by the α3(IV)α4(IV)α5(IV) and the [α5(IV)]_2_α6(IV) isoforms of collagen IV. Anti-α5(IV) antibody, raised against the NC1 domain of the molecule, as expected labelled the bulk of the ILM (Fig. 2g–i) which is known to be formed prevalently by the α3(IV)α4(IV)α5(IV) isoform [6, 33]. The same antibody, however, did not stain iERMs. In contrast, anti-collagen IV and anti-collagen I antibodies, already employed on de-plasticized sections (Figs. 1 and 2), strongly labelled iERMs, whereas the ILMs were stained only on their vitreal side (Fig. 2g–i), thus confirming results obtained on Epon-embedded samples. As there was no overlapping fluorescence between the anti-collagen IV and the anti-α5(IV) antibodies, the former antibody likely recognized the [α1(IV)]_2_α2(IV) isoform which is the only isoform that does not contain the α5(IV) chain. Hence, different isoforms of collagen IV occupied two diverse domains within the ILM, the [α1(IV)]_2_α2(IV) isoform being on the vitreal half and an α5(IV)-containing isoform (presumably the α3(IV)α4(IV)α5(IV)) on the retinal half. Interestingly, an unstained stripe intervened between the two layers immunoreactive with the anti-collagen IV and the anti-α5(IV) antibodies (Fig. 2g–i).

**Fig. 3:**
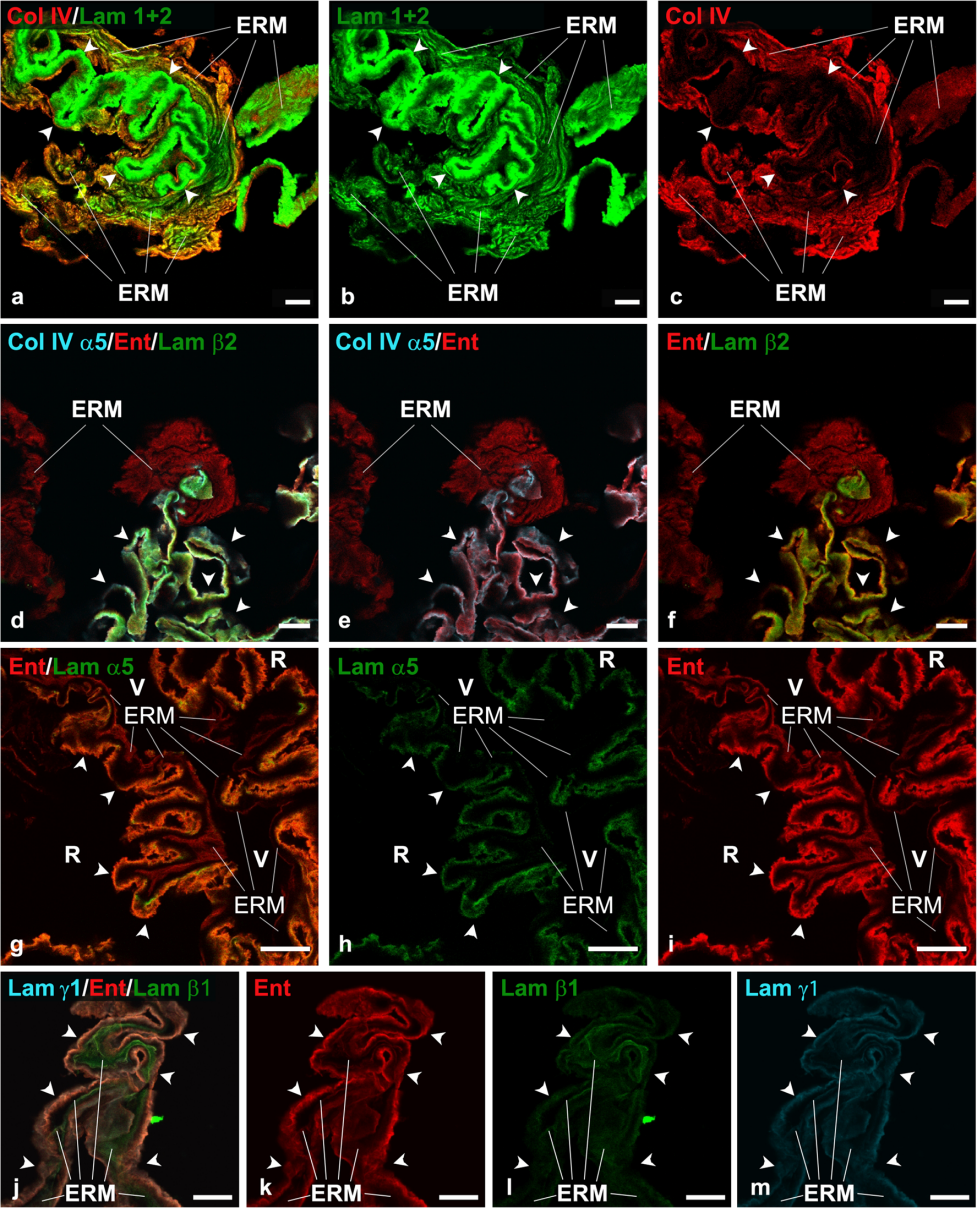
Chain-specific laminin expression in ILM and iERMs. **a–c** Idiopathic ERM/ILM double labelled with anti-type IV collagen (red) and anti laminin 1+2 (green) antibodies. The iERM expresses both antigens. The ILM (arrowheads) shows an exuberant immunoreactivity for the anti-laminin 1+2 antibody. **d–f** Idiopathic ERM/ILM triple labelled with anti-α5(IV) (cyan), anti-entactin (red), and anti-laminin β2 (green) antibodies. The α5 chain of collagen IV and the laminin β2 are expressed exclusively in the ILM (arrowheads). Entactin, in contrast, is expressed either in the iERM or in the ILM. **g–i** Idiopathic ERM/ILM double labelled with anti-entactin (red) and anti-laminin α5 (green) antibodies. Whereas laminin α5 is present almost exclusively in the ILM (arrowheads), entactin is expressed in both membranes. V vitreal side of the iERM, R retinal side of the ILM. **j–m** Idiopathic ERM/ILM triple labelled with anti-laminin γ1 (cyan), anti-entactin (red), and anti-laminin β1 (green) antibodies. Though with a different degree of intensity, all antigens are expressed either in the ILM (arrowheads) or in the iERM. Entactin and the laminin γ1 are prevalently located in the ILM, whereas the laminin β1 fluorescence is more evident in the iERM. ERM epiretinal membrane. **a–f** Magnification bars = 10 μm. **g**–**m** Magnification bars = 20 μm

Laminins are trimeric molecules composed of the assembling of α-, β- and γ-chains. Alpha-, β-, and γ-chains have several variants. In contrast to the early nomenclature consisting of a progressive number (laminin 1, laminin 2, laminin 3, and so on…), each laminin isoform is currently identified by its chain composition (α1β1γ1 laminin is referred to as laminin 111 and corresponds to laminin 1 of the former designation). To identify laminin isoforms in our samples, we employed a battery of antibodies. The main results are reported in Table 1. In summary, we observed that ILMs were strongly immunoreactive with anti-laminin 1 + 2 (Fig. 3a–c), anti-laminin β2 (Fig. 3d–f), anti-laminin α5 (Fig. 3g–i), anti-laminin β1, and anti-laminin γ1 antibodies (Fig. 3j–m), whereas a weak and focal labelling on the retinal side was achieved only occasionally with the anti-laminin α2 antibody (Online Resource 2a-c). On the other hand, iERMs were consistently labelled with the anti-laminin 1 + 2 (Fig. 3a–c), anti-laminin β1, and anti-laminin γ1 antibodies (Fig. 3j–m; Online Resources 2d-f). A very weak and inconstant fluorescence was occasionally observed with anti-laminin α5 antibody (Fig. 3g–i).

**Table 1:** Anti-laminin antibodies employed in the study and their reactivity with the ILMs and the ECM of ERMs

Antibody	Brand	Code	ILM	ERM
Laminin 1 + 2	Abcam	Ab7463	+	+
Laminin-2 (α2 chain)	Gibco SRL	12076-014	Focally + on the retinal side	-
Laminin (α4 chain)	Santa-Cruz	sc-130541	-	-
Laminin (α5 chain)	Novus Biologicals	NBP2-42391	+	Mostly -
Laminin (β1 chain)	Santa-Cruz	sc-33709	+	+
Laminin (β2 chain)	Santa-Cruz	sc-59980	+	-
Laminin (β3 chain)	Santa-Cruz	Sc-133178	-	-
Laminin (γ1 chain)	Chemicon	MAB1920	+	+
Laminin-5 (γ2 chain)	Dako	M7262	-	-
Laminin-5 (γ2 chain)	Chemicon	MAB19562	-	-

**Fig. 4:**
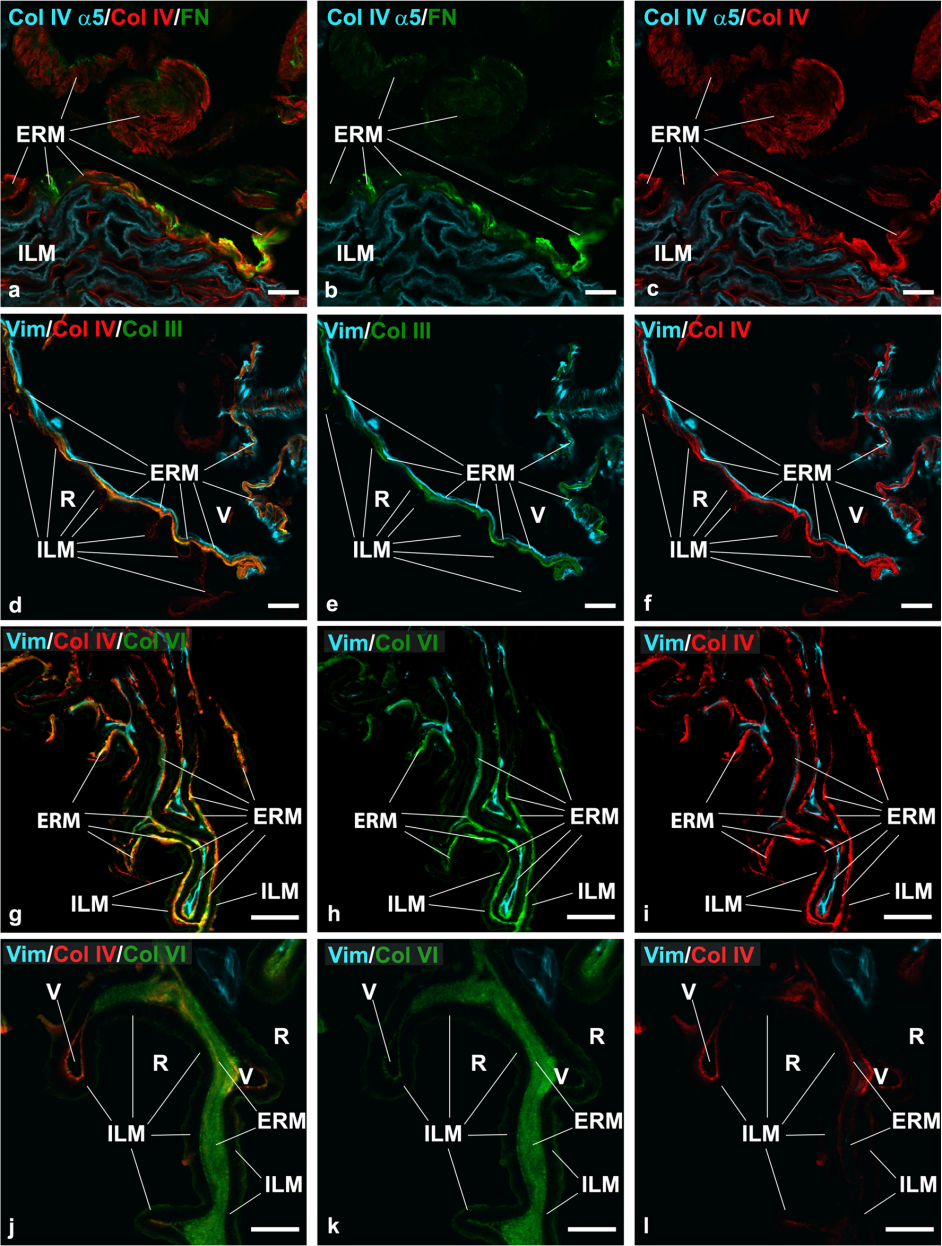
Expression of fibronectin, collagen III, and collagen IV in iERM and ILM. **a–c** Idiopathic ERM/ILM triple labelled with anti-α5(IV) (cyan), anti-collagen IV ([α1(IV)]_2_α2(IV) isoform) (red), and anti-fibronectin (green) antibodies. The iERM expresses both fibronectin and the [α1(IV)]_2_α2(IV) isoform of collagen IV though their distribution co-localizes only focally. The α5 chain of collagen IV is restricted to the ILM. Note the different localization of the [α1(IV)]_2_α2(IV) isoform of collagen IV and the α5 chain of same collagen in the ILM. **d-f)** Idiopathic ERM/ILM triple labelled with vimentin (cyan), anti-collagen IV ([α1(IV)]_2_α2(IV) isoform) (red), and anti-collagen III (green) antibodies. Collagens III and IV co-localize perfectly in the iERM. In contrast, the ILM, which is well visible where the iERM is detached, shows only the usual weak immunoreactivity for the [α1(IV)]_2_α2(IV) isoform of collagen IV on its vitreal rim. Note how cells are gathered on the vitreal side of the ERM and the relationships occurring between the ILM and the ERM with the latter which is shorter and bridges more distant points of the ILM. R retinal side, V vitreal side. **g–l** Idiopathic ERM/ILM triple labelled with vimentin (cyan), anti-collagen IV ([α1(IV)]_2_α2(IV) isoform) (red), and anti-collagen VI (green) antibodies. The [α1(IV)]_2_α2(IV) isoform of collagen IV and collagen VI co-localizes in the iERM though there are areas labelled exclusively anti-collagen IV or anti-collagen VI antibodies. In general, however, collagen VI seems more represented (particularly in **j**–**l**). The ILM is labelled on both the retinal and the vitreal edges. Where the iERM is detached from the ILM, collagen VI fluorescence is visible on the retinal edge of the ILM co-localizing perfectly with the [α1(IV)]_2_α2(IV) isoform of collagen IV. R retinal side, V vitreal side. ILM inner limiting membrane, ERM epiretinal membrane. **a**–**i** Magnification bars = 20 μm. **j**–**l** Magnification bars = 10 μm

We also checked the presence of entactin, fibronectin, collagen III, and collagen VI either in iERMs or in ILMs. Results are summarized in Table 2. Entactin was expressed in both membranes, though its labelling was stronger in ILMs. In ILMs, entactin co-localized with the anti-α5(IV) and anti-laminin β2 antibodies (Fig. 3d–f); anti-laminin α5 (Fig. 3g–i), anti-laminin β1, and anti-laminin γ1 antibodies (Fig. 3j–m); and with anti-laminin 1 + 2 antibody (data not shown). In iERMs, entactin co-localized with anti-laminin β1 and anti-laminin γ1 antibodies (Fig. 3j–m) and with anti-laminin 1 + 2 antibody (data not shown). Fibronectin and collagen III were not found associated with ILMs. However, antibodies to both ECM proteins double-labelled with the anti-collagen IV antibody in iERMs (Fig. 4a–f) though fibronectin/collagen IV co-localization was only partial (Fig. 4a–c). In contrast, there was a more widespread match between collagen III and collagen IV distribution (Fig. 4d–f). Anti-collagen VI antibody, on the other hand, generated a very strong fluorescence in iERMs where it partially co-localized with anti-collagen IV antibody (Fig. 4g–l). Collagen VI could be also observed either on the vitreal edge of ILMs, where it perfectly overlapped [α1(IV)]_2_α2(IV) collagen immunofluorescence, or on the retinal side, where it showed a less strong immunoreactivity (Fig. 4j–l).

**Table 2:** Anti-ECM protein (excluding laminin chains) antibodies employed in the study and their reactivity with the ILMs and the ERMs

Antibody	Brand	code	ILM	ERM
Type I collagen (α1 chain)	Novus Biologicals	NB600-408	+ on the vitreal edge	+
Type III collagen	Abcam	ab7778	−	+
Type IV collagen ([α1]_2_α2 isoform)	Southern Biotech	1340-01	+ on the vitreal edge	+
Type IV collagen (α5 chain)	Chondrex	7077	+	−
Type VI collagen	Abcam	Ab6588	+ on the vitreal and retinal edges	+
Entactin	R&D Systems	AF2570	+	+
Fibronectin	GeneTex	GTX112794	−	+

## Discussion

The morphology of iERMs has been studied quite extensively [20, 22, 23, 34]. However, the variations occurring in the relationships between iERMs and the ILM received little attention [20]. The unusual technique that we employ, immunofluorescence and confocal microscopy on semithin epon-embedded sections [17, 23], produces high-quality images that allow the study of details that probably have gone unnoticed so far. In this way, we have observed that the relationships between iERMs and their associated ILMs vary greatly: from very thin iERMs, just covering the ILM, to iERMs that, slowly gliding over the ILM, induce the latter to form large loops [20]. In some cases, the folds of the ILM under the iERM do not appear as empty loops but they are filled by the ECM. Some specimens, showing a looser arrangement of the ECM, are suggestive for a possible mechanism that could explain why some iERMs generate macular puckers. In such samples, folding of the ILM appears as the result of the deposition of a series of progressively shorter ECM lamellae. In this way, the more the iERM grows thicker, the more the ECM lamellae could produce a progressively stronger tangential traction to the underlying ILM. If this were true, the wrinkling of the retinal surface, characteristic of macular puckers, could be due not only to the progressive traction exerted by myofibroblasts [35–37] but also to the peculiar pattern of ECM deposition. This sort of slipping phenomenon seems operating even within the iERM itself as a straight cell layer located on the vitreal side can be caught lying over folds of the underlying ECM. This result could be produced by their contraction or just by their moving on the ECM or by a combination of the two factors. If adherens-type intercellular junctions, which have been reported as a common finding in iERM cells [38], maintain the cell layer cohesive enough, a different moving behaviour of groups of cells in diverse areas of the iERM could induce the folding of the ECM exactly as a carpet, not adherent to the floor, can slip under the feet of a walking man.

It is evident that a mechanism like this probably depends on the ECM proteins of the iERMs, on their relationship, and on their adhesiveness to the ILM. A precise knowledge of the ECM proteins exposed on the vitreal side of the ILM is also important as they can provide a link for strongly adherent iERMs. For this reason, we undertook an in-depth immunofluorescence study over the ECM proteins expressed in iERMs and in their associated ILMs.

As far as the ILM is concerned, our results confirm and expand previously published findings [39]. In particular, we assessed that the ILM shows a bilaminar composition which is testified by the distribution of different collagen isoforms. The retinal half of the ILM contains an α5(IV)-chain containing collagen, very likely the α3(IV)α4(IV)α5(IV) isoform as it is the major component of the ILM [6, 33]. In contrast, on its vitreal side, the ILM shows a constant immunoreactivity with the anti-collagen IV antibody that recognizes the [α1(IV)]_2_α2(IV) isoform. A minor amount of the [α1(IV)]_2_α2(IV) isoform in the ILM has been previously reported [40], though its precise location was still undetermined. Our results show that the two isoforms of collagen IV occupy different domains in the ILM with an intervening unstained stripe. This is in accordance with the specificity of the antibody employed to detect the α5(IV) as it labels the NC1 domain of the molecule that is located on the retinal side of the ILM [39]. The unstained stripe of tissue between the two immunofluorescent layers of collagen IV, therefore, likely corresponds to the 7S domain of the α3(IV)α4(IV)α5(IV) isoform which is adjacent and does not overlap with the NC1 domain as previously reported [39].

The fluorescence obtained with the antibody against the NC1 domain of α5(IV), on the other hand, co-localizes with laminin. This confirms previous results that locate laminin on the retinal side of the ILM [39]. We have found that the ILM contains laminin α1 (as a component of laminin 111), laminin α5, laminin β1, laminin β2, laminin γ1, and laminin α2. In this respect, a certain ambiguity exists in previously published researches as far as the human ILM is concerned where only laminin γ1 was detected in adult samples [41]. In contrast, a pattern of expression very similar to ours has been observed in the ILM of the developing eye [41]. Whereas laminin β3, laminin γ2, and laminin γ3 have never been reported in human ILM, proteomic analysis has detected minor amounts of the α1, α2, α3, and α4 chains [40]. Though we actually did not detect laminin α3 and laminin α4, proteomic corroborates our findings on laminin α2 and laminin 111 whose expression in the ILM is controversial [41, 42].

The combination of the immunodetected laminin chains, therefore, can easily account for the presence in the adult human ILM of the following trimers: laminin 111 (former laminin 1), laminin 521 (former laminin 11), and laminin 211 (former laminin 2). We can rule out the occurrence of laminin 213 (former laminin 12), laminin 332 (former laminin 5), laminin 323 (former laminin 13), laminin 423 (former laminin 14), laminin 523 (former laminin 15), and laminin 522 as, by all accounts, the ILM lacks at least one of their constituting chains (β3, γ2 and γ3). On the other hand, even though we did not detect laminin α4, the presence of laminin 421 (former laminin 9) cannot completely excluded as low amounts of laminin α4 were found by proteomic [40]. It is possible that the quantity of laminin α4 in ILMs is under the threshold of detection by immunofluorescence, at least with the antibody that we employed. Finally, laminin 121 (former laminin 3), laminin 221 (former laminin 4), laminin 311 (former laminin 6), laminin 321 (former laminin 7), and laminin 511 (former laminin 10) are isoforms that cannot be ruled out a priori because we did not tested the α3 chain or because of possible recombination of the detected chains in alternative trimers.

The retinal side of the ILM also contains entactin. Actually, entactin fluorescence matches the immunoreactivity for the NC1 domain of α5(IV) and for laminins. Such precise co-localization is the result of the interactions occurring between entactin on one side, and collagen IV and laminin on the other side. Entactin bridges collagen IV and laminin lattices in all basement membranes, including ILM, binding collagen IV close to its NC1 domain [43].

The [α1(IV)]_2_α2(IV) isoform of collagen IV, located on the vitreal side of the ILM, appears to co-localize with collagens I and VI. Interestingly, collagen VI is known to bind with high affinity to collagen IV [44]. This property could account for its presence on both sides of the ILM where different collagen IV isoforms are located. In addition, collagen VI can bind directly to itself and to collagen I [44, 45]. Binding to collagen I can be also mediated by interactions with fibronectin that can contribute to glue together collagens I, VI, and IV [44, 46]. This notation is important as collagens I, IV, and VI can provide a mean to promote iERM adherence to the ILM. Collagen VI, on the other hand, provides an anchoring site for several cell receptors, like α1β1 and α2β1 integrins [47]. In general, as collagen-binding integrins may interact with collagens I, IV, and VI [48], collagens exposed on the vitreal side of the ILM can provide attachment sites for cells of the ERM. In this respect, the α2β1 collagen-binding integrin has been shown in iERMs [49]. Many other integrins, known to bind to collagens (like α1β1, α9β1, α10β1, and α11β1), however, to our knowledge, have never been tested in iERMs..

Idiopathic ERMs express collagens I, III, IV, and VI; fibronectin; and laminin as previously reported [17, 22, 23]. As far as collagen IV is concerned, the isoform detected in iERMs should be the [α1(IV)]_2_α2(IV). This can be inferred by the lack of staining with the anti-α5(IV) antibody that excludes all the other known collagen IV isoforms. Collagen IV in ERMs co-localizes with collagen I, III, and VI. This is not unexpected as it has been shown that collagen IV in iERMs is not restricted to basement membranes [23]. On the other hand, the reverse is not always valid as several areas of the ERMs contain collagens I, III, and VI without the presence of collagen IV. Yet, the constant co-localization of collagen IV with the anti-laminin 1 + 2 antibody and not with the anti-laminin α2 antibody suggests that the isoform detected in iERMs is with all probability laminin 111. The only other laminin highly expressed in the ILM, laminin 521, is certainly not present as the anti-laminin β2 antibody fails to stain iERMs. In contrast, the expression of laminin β1 and laminin γ1 is consistent with the presence of laminin 111 in iERMs. On the other hand, the weak signal obtained sometimes with the anti-laminin α5 antibody suggests the possible expression of minor amounts of laminin 511.

The iERMs constantly express entactin, another protein characteristic of basement membranes. Entactin co-localizes with laminin and, therefore, with collagen IV as well. The presence of almost all the major constituents of basement membranes suggests the possibility that iERM development could be the result of a deranged attempt to organize a basement membrane. The reason why these molecules, that ordinarily self-assemble [50], fail to produce a real basement membrane is uncertain. It is possible that the concomitant presence of other collagens, like collagen I and III, may interfere in the process.

It is worthwhile to note that the ILM has a composition similar to the glomerular basement membrane in the kidney. The major constituents of both membranes in the adult are laminin 521 and the isoform α3(IV)α4(IV)α5(IV) of collagen IV though the histogenetic process of both membranes begins with the deposition of laminin 111 and collagen IV [α1(IV)]_2_α2(IV) [51, 52]. In Alport syndrome, a genetic nephritic disease accompanied by ocular manifestations and caused by mutations involving *COL4A5*, *COL4A3*, or *COL4A4* genes, TGFβ1 plays a central role in inducing an abnormal deposition of foetal laminin 111 and collagen [α1(IV)]_2_α2(IV) in the glomerular basement membrane [53, 54]. Interestingly, even the development of iERMs appears driven by TGFβ1 expression [16, 17] and, likewise the glomerular basement membrane, iERMs express the same collagen IV and laminin isoforms that are produced early in foetal development.

In conclusion, we have proposed a possible mechanism for the generation of some macular puckers based on the structural arrangement of the ECM of iERMs. In addition, we confirmed that iERMs contain collagen I, fibronectin, and collagen III, and we have demonstrated that iERMs express laminin 111, collagen IV [α1(IV)]_2_α2(IV), and entactin. We have also shown the bilaminar structure of the ILM, as far as collagen IV isoforms are concerned, and we could define the precise distribution of some of its components from the retinal to the vitreal side.

## Supplementary information

Online Resource 1 One ILM removed for a macular hole not complicated by the presence of an iERM was double-labelled with anti-collagen I (green) and anti-collagen IV (red) antibodies. Both antigens stain the vitreal edge of the ILM confirming that they are constitutive components of the ILM. V vitreal side; R retinal side; magnification bars = 20 μm (JPG 215 kb)

Online Resource 2 **a-c)** One ERM with associated ILM was double-labelled with anti-laminin α2 (green) and anti-collagen IV (red) antibodies. The field shows a tract of ILM dissociated from the ERM. Laminin α2 and collagen IV ([α1(IV)]2α2(IV) isoform) are located on the opposite sides of the ILM. V = vitreal side; R = Retinal side; Magnification bars 10 μm. **d-f).** One ERM was double-labelled with anti-laminin β1 (green) and anti-laminin γ1 (red) antibodies. The two laminin chains co-localize in the ERM. Magnification bars = 50 μm (JPG 1055 kb)

## Data Availability

Original images acquired at the confocal microscope are available at the corresponding author’s address upon reasonable request.
