# Supplementary figures and images for: The extracellular matrix complexity of idiopathic epiretinal membranes and the bilaminar arrangement of the associated internal limiting membrane in the posterior retina
Source: Graefes Arch Clin Exp Ophthalmol. 2021 Mar 24;259(9):2559–71. doi: 10.1007/s00417-021-05156-6 (PMC8380574; doi:10.1007/s00417-021-05156-6)

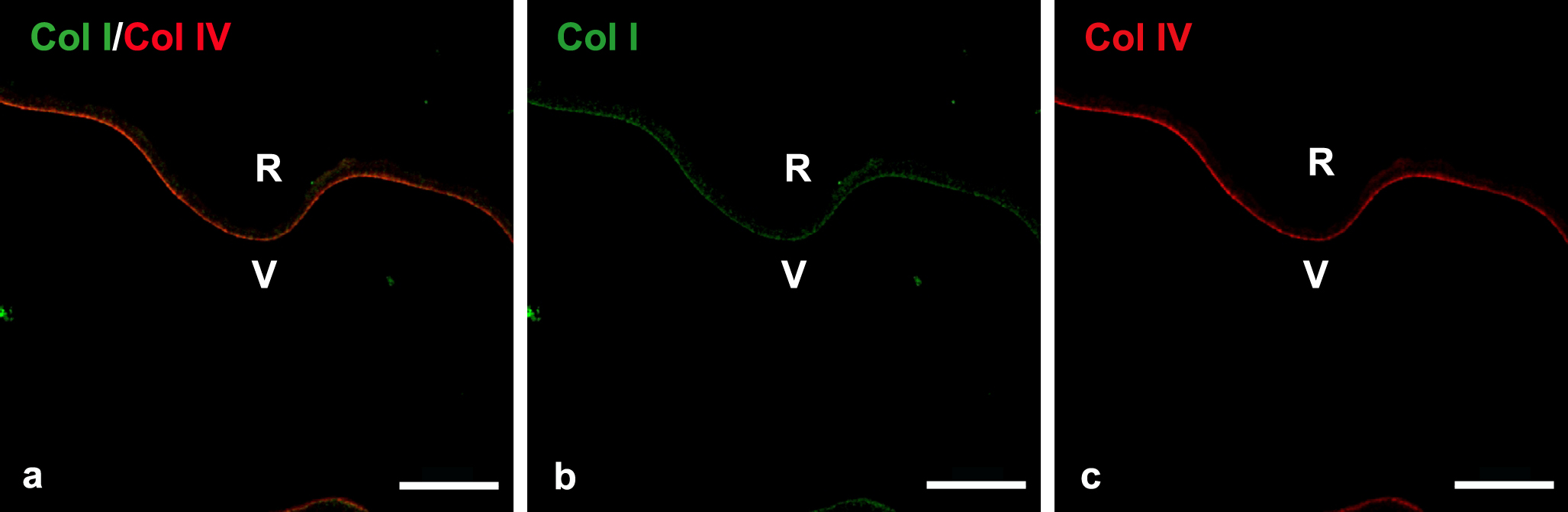

Supplement: Supplementary file 1 — One ILM removed for a macular hole not complicated by the presence of an iERM was double-labelled with anti-collagen I (green) and anti-collagen IV (red) antibodies. Both antigens stain the vitreal edge of the ILM confirming that they are constitutive components of the ILM. V vitreal side; R retinal side; magnification bars = 20 μm (JPG 215 kb) [file 417_2021_5156_MOESM1_ESM.jpg]

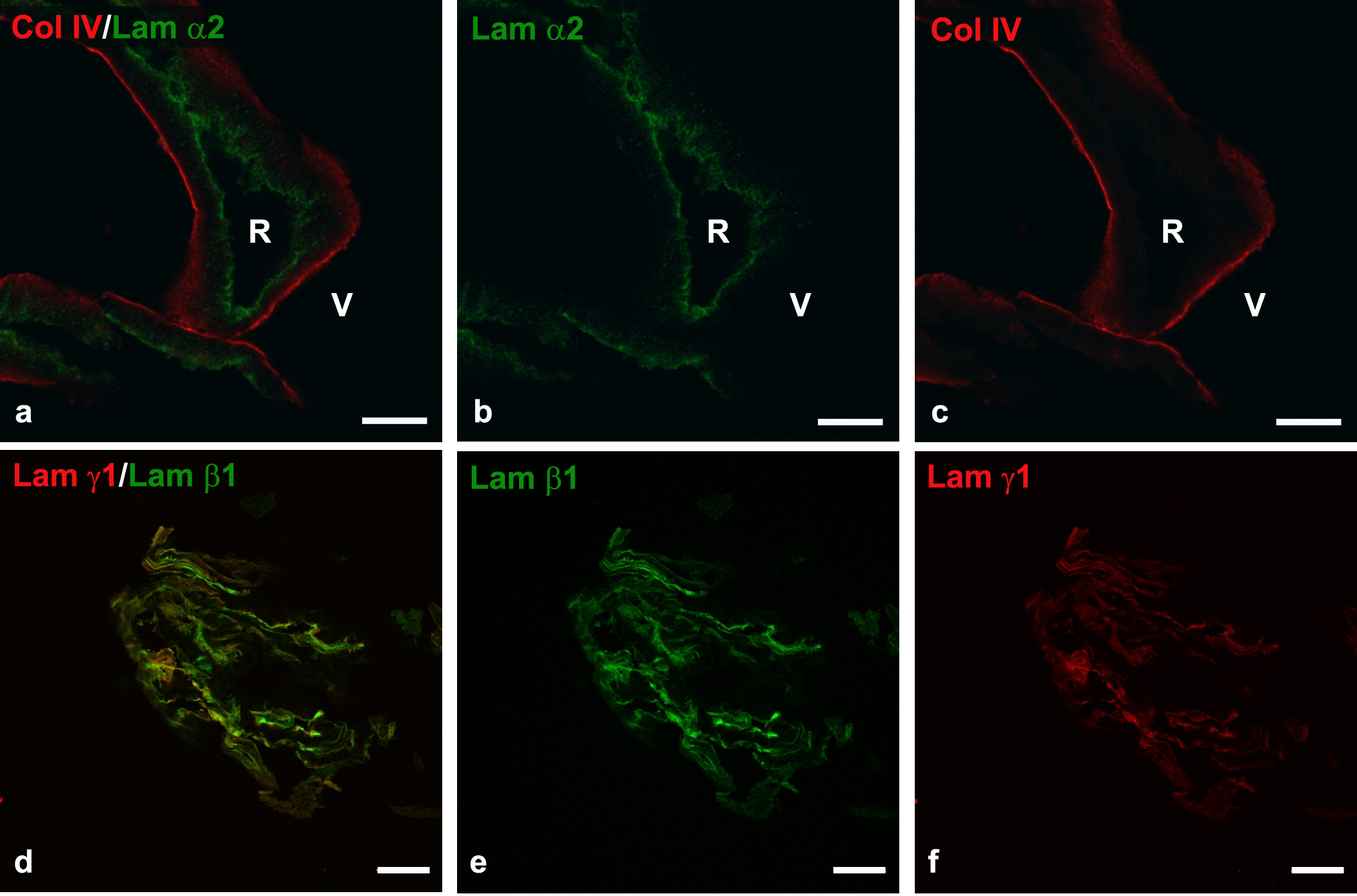

Supplement: Supplementary file 2 — a-c) One ERM with associated ILM was double-labelled with anti-laminin α2 (green) and anti-collagen IV (red) antibodies. The field shows a tract of ILM dissociated from the ERM. Laminin α2 and collagen IV ([α1(IV)]2α2(IV) isoform) are located on the opposite sides of the ILM. V = vitreal side; R = Retinal side; Magnification bars 10 μm. d-f). One ERM was double-labelled with anti-laminin β1 (green) and anti-laminin γ1 (red) antibodies. The two laminin chains co-localize in the ERM. Magnification bars = 50 μm (JPG 1055 kb) [file 417_2021_5156_MOESM2_ESM.jpg]
